# Reply: Smoking and hepatoblastoma: confounding by birth weight?

**DOI:** 10.1038/sj.bjc.6601144

**Published:** 2003-07-29

**Authors:** D Pang, J M Birch

**Affiliations:** 1 Cancer Research UK, University of Manchester, Paediatric & Familial Cancer Research Group, Royal Manchester Children's Hospital, Stancliffe, Manchester M27 4HA, UK

**Sir**,

The question asked by Spector and Ross is interesting because birth weight is important in understanding parental smoking in relation to hepatoblastoma. Two aspects may be worth clarifying: (a) whether the association between parental smoking and hepatoblastoma is due to the confounding effect of low birth weight of the index child, and (b) whether the association between parental smoking and hepatoblastoma is due to an indirect effect of smoking through low birth weight rather than the direct effect of smoking on fetal liver.

Our original analysis of parental prenatal smoking based on self-reported data collected from parents of children with hepatoblastoma and parents of population controls, showed the following: maternal smoking, OR=2.68 (*P*<0.05); both parents smoking, OR=4.74 (*P*<0.01); maternal smoking in cases diagnosed at the median age for hepatoblastoma or older, OR=12.02 (*P*<0.01) (Pang *et al*, 2003). We have now repeated the analyses adjusting for low birth weight as well as parental age and deprivation. Low birth weight was defined as less than 2000 g (Moore and Persaud, 1993) and treated as 0/1 binary variable. Results were obtained as follows: maternal smoking OR=2.50, both parents smoking OR=4.97, older children OR=12.66, in all cases *P*<0.05. We may conclude, therefore, that low birth weight does not explain the observed association between parental smoking and hepatoblastoma.

We are not convinced that the association reflects an indirect effect through low birth weight. Hepatoblastoma is an embryonal tumour which most likely has a fetal origin and prenatal risk factors probably play a more important role than postnatal risk factors. The case for this may be made as follows: first, in some children, hepatoblastoma is evident at birth or a few months after birth (median age at diagnosis is about 1 year). Second, histopathological examination shows striking similarities in size and configuration between the developing fetal liver cells and the fetal epithelial-type cells of hepatoblastoma. Third, the tumour's ability to synthesise alpha-fetoprotein (AFP) points to a fetal origin. AFP, a major serum protein synthesised by fetal liver cells, is also found in children with hepatoblastoma. Fourth, hepatoblastoma, like Wilms’ tumour, is associated with Beckwith–Wiedemann syndrome and hemihypertrophy, suggesting gestational oncogenic events (DeBaun and Tucker, 1998).

Furthermore, previous studies have shown that carcinogenic metabolites of tobacco smoke can cross the placenta, the natural barrier protecting the fetus from foreign substances, and the fetal liver, as a detoxification organ, is immature and vulnerable to carcinogens. A causal association with parental smoking is also supported by the finding of G to T transversions in the TP53 gene in hepatoblastoma (Olivier *et al*, 2002), a type of mutation that is typical of smoking-induced carcinoma of the lung (Pfeifer *et al*, 2002).

In conclusion, our study suggests that parental smoking may play a role in the development of hepatoblastoma. However, as we stated, the finding may have arisen by chance since we carried out many statistical comparisons. Further studies of parental smoking, birth weight and other potential complications in relation to hepatoblastoma would be helpful. As only about 15 cases of hepatoblastoma occur each year in the UK and 100 cases a year in the US, an international effort would be required.
